# ABHD15 regulates adipose tissue lipolysis and hepatic lipid accumulation

**DOI:** 10.1016/j.molmet.2019.05.002

**Published:** 2019-05-06

**Authors:** Jacqueline Stöckli, Armella Zadoorian, Kristen C. Cooke, Vinita Deshpande, Belinda Yau, Gaia Herrmann, Melkam A. Kebede, Sean J. Humphrey, David E. James

**Affiliations:** 1 Charles Perkins Centre, School of Life and Environmental Sciences, University of Sydney, Sydney, NSW, 2006, Australia; 2 Sydney Medical School, University of Sydney, Sydney, NSW, 2006, Australia

**Keywords:** Lipolysis, Adipocytes, Insulin action, Fatty acid metabolism, β-adrenergic receptor agonist

## Abstract

**Objective:**

Insulin suppresses adipose tissue lipolysis after a meal, playing a key role in metabolic homeostasis. This is mediated via the kinase Akt and its substrate phosphodiesterase 3B (PDE3B). Once phosphorylated and activated, PDE3B hydrolyses cAMP leading to the inactivation of cAMP-dependent protein kinase (PKA) and suppression of lipolysis. However, several gaps have emerged in this model. Here we investigated the role of the PDE3B-interacting protein, α/β-hydrolase ABHD15 in this process.

**Methods:**

Lipolysis, glucose uptake, and signaling were assessed in ABHD15 knock down and knock out adipocytes and fat explants in response to insulin and/or β-adrenergic receptor agonist. Glucose and fatty acid metabolism were determined in wild type and ABHD15^−/−^ littermate mice.

**Results:**

Deletion of ABHD15 in adipocytes resulted in a significant defect in insulin-mediated suppression of lipolysis with no effect on insulin-mediated glucose uptake. ABHD15 played a role in suppressing PKA signaling as phosphorylation of the PKA substrate Perilipin-1 remained elevated in response to insulin upon ABHD15 deletion. ABHD15^−/−^ mice had normal glucose metabolism but defective fatty acid metabolism: plasma fatty acids were elevated upon fasting and in response to insulin, and this was accompanied by elevated liver triglycerides upon β-adrenergic receptor activation. This is likely due to hyperactive lipolysis as evident by the larger triglyceride depletion in brown adipose tissue in these mice. Finally, ABHD15 protein levels were reduced in adipocytes from mice fed a Western diet, further implicating this protein in metabolic homeostasis.

**Conclusions:**

Collectively, ABHD15 regulates adipocyte lipolysis and liver lipid accumulation, providing novel therapeutic opportunities for modulating lipid homeostasis in disease.

## Highlights

• Insulin was unable to suppress lipolysis in the absence of ABHD15 in adipocytes *in vitro*, *ex vivo* and in mice *in vivo.*
• The lipolysis defect was associated with defective signalling via protein kinase A and its substrate Perilipin-1.
• The defect was specific for lipolysis with no impairment in insulin signalling or insulin-stimulated glucose uptake.
• Deletion of ABHD15 caused a significant increase in fatty acid deposition in liver in response to stress.

## 1 Introduction

Insulin coordinates several metabolic processes in adipose tissue that play a key role in metabolic regulation throughout the body. In addition to activating glucose transport via translocation of the glucose transporter GLUT4 and the subsequent intracellular metabolism of glucose, insulin also has a potent inhibitory effect on the release of free fatty acids from the fat cell, a process called lipolysis. Lipolysis is induced in response to starvation via catecholamines and other agonists and this is suppressed in response to a meal via insulin. Catecholamine-mediated induction of lipolysis involves activation of the β-adrenergic receptor leading to elevated cAMP and activation of cAMP-dependent protein kinase (PKA). PKA phosphorylates several proteins involved in lipolysis, including the lipid droplet proteins Perilipin-1 (PLIN1) and Hormone Sensitive Lipase (HSL) [1]. PLIN1 phosphorylation results in its release from CGI-58, leading to the formation of an active complex comprising Adipose Triglyceride Lipase (ATGL) and its co-activator CGI-58 that initiates the first step in triglyceride breakdown. Phosphorylation of HSL activates and recruits the lipase responsible for the subsequent step in triglyceride lipolysis that ultimately results in the complete hydrolysis of triglycerides into glycerol and three fatty acids.

The mechanism by which insulin suppresses lipolysis in adipocytes is less well understood, and several gaps have recently emerged. It was initially thought that insulin's effects on lipolysis were mediated via Akt-dependent phosphorylation and activation of PDE3B leading to hydrolysis of cAMP thereby inactivating PKA [2]. Both Akt and PDE3B are essential for insulin's anti-lipolytic actions as insulin mediated suppression of lipolysis is abolished in the presence of Akt kinase inhibitors [3] or in PDE3B^−/-^ adipocytes [4], [5]. However, while both PDE3B and Akt are essential for this process, neither PDE3B activity (cAMP hydrolysis) nor PDE3B phosphorylation by Akt are necessary for insulin's anti-lipolytic effects [5], [6]. This is because PDE3B mutants in which both of these functions were disarmed can rescue the effects of insulin on lipolysis in PDE3B^−/-^ adipocytes [5]. These data suggest an important role for other yet unidentified lipolysis regulators.

ABHD15 displays several properties implicating it as having an important role in lipolysis. It is a member of the α/β-hydrolase family of proteins that comprises 120 members and includes several other lipolytic regulatory proteins, such as HSL and the ATGL coactivator CGI-58 (also called ABHD5) [7]. ABHD15 interacts with and may stabilize PDE3B, as its loss results in reduced PDE3B levels [8]. ABHD15 expression is induced upon adipocyte differentiation and it is strongly phosphorylated in response to insulin [9], [10].

Here we show that insulin-stimulated suppression of lipolysis is blocked in the absence of ABHD15 in adipocytes. Deletion of ABHD15 also led to defective suppression of PKA-induced PLIN1 phosphorylation and reduced levels of PDE3B. Consistent with this, ABHD15^−/−^ mice displayed elevated circulating free fatty acids upon fasting and in response to insulin. Furthermore, ABHD15^−/−^ mice showed increased liver triglycerides upon β-adrenergic receptor activation, compared to wild type littermates, indicating that stress-induced lipid accumulation in liver is markedly exacerbated when lipolysis is unrestricted. These studies provide new insights into the regulation of adipocyte lipolysis.

## 2 Materials and methods

### 2.1 Reagents, antibodies and constructs

Tissue culture reagents were purchased from Life Technologies (Thermo Fisher Scientific), and chemicals were obtained from Sigma unless otherwise indicated. BSA was purchased from Bovogen, and protease inhibitors were from Roche. Pierce BCA Protein Assay and TCEP (Tris(2-carboxyethyl)phosphine) were obtained from Thermo Fisher Scientific.

Antibodies were purchased from Cell Signaling Technology (pThr642-AS160, HSL, pSer660-HSL, pThr308-Akt, pSer473-Akt, pThr246-PRAS40, ATGL, IgG), Vala Sciences (pSer522-PLIN1), Sigma (tubulin), and Santa Cruz Biotechnology (PDE3B, 14-3-3). Two antibodies against ABHD15 were used in this study; one was kindly provided by Gus Lienhard [10], and one was purchased from ProteinTech. Secondary antibodies were purchased from Li-Cor (IRDye 700 and 800), Jackson Immunoresearch (HRP-anti-mouse and rabbit), and Abcam (HRP-anti-sheep).

Human cDNA of ABHD15 (Open Biosystems) was amplified and cloned into pWZL-NEO retroviral plasmid. SV40-LT was transferred from pBABE-PURO (Addgene) into pBABE-HYGRO via restriction enzyme cloning. Control shRNA against luciferase [11] was cloned into pBABE-PURO and shRNA against ABHD15 was kindly provided by Gus Lienhard [8]. PDE3B siRNA was purchased from Sigma.

### 2.2 Cell culture, retrovirus, siRNA transfection

Brown preadipocytes were isolated as previously described [12] from newborn mice produced from an ABHD15^+/−^ breeding pair. ABHD15^−/−^ and wild type cells derived from littermate newborn mice were immortalized by infection with hygromycin resistant retroviral vector pBABE encoding SV40-LT, followed by selection using 100 μg/ml hygromycin. Cells were differentiated into adipocytes as previously described [13]. Differentiated adipocytes were maintained in DMEM+10% FBS from day 6 onwards and experiments were undertaken on days 9–10 post differentiation.

3T3-L1 fibroblasts were obtained from Howard Green [14], cultured in DMEM containing 10% FBS and GlutaMAX and differentiated into adipocytes as previously described [15].

PlatE cells [16] grown in 10 cm dish were transiently transfected using Lipofectamine 2000 (Life Technologies) according to manufacturer's instructions with retroviral vectors pBABE or pWZL-NEO. Medium was replaced the next day with 6 ml medium per dish. Virus-containing medium was collected after 2 days (1 d at 37 °C + 1 d at 32 °C), followed by filtration using a 0.45-micron filter and used immediately for infection or stored at −80 °C.

3T3-L1 fibroblasts were infected with puromycin resistant retroviral vector pBABE encoding shRNA against ABHD15 or control shRNA against luciferase, via overnight infection in the presence of 4 μg/ml polybrene, followed by selection with 2 μg/ml puromycin. ABHD15 re-expression was achieved by infecting 3T3-L1 and brown preadipocytes with geneticin resistant retroviral vector pWZL-NEO encoding human ABHD15 (or empty vector as control), followed by selection with 0.8 mg/ml geneticin (Life Technologies).

3T3-L1 adipocytes were electroporated with 200 nM control or PDE3B siRNA (sense 5′-GAUGUAAAUAGUAACGGUATT-3′) at day 7 of differentiation as previously described [17]. Cells were subjected to assessment of lipolysis and immunoblotting 4–5 d after siRNA transfection.

### 2.3 Lipolysis assay and glucose uptake in adipocytes

Lipolysis assay was performed in 3T3-L1 and brown adipocytes grown in 24 or 12-well plates as previously described with 2–3 technical replicates per data point [3]. Briefly, adipocytes were serum starved in DMEM containing 0.2% BSA for 2 h prior to incubation of cells for 1 h in Krebs–Ringer Phosphate (KRP) buffer (0.6 mM Na_2_HPO_4_, 0.4 mM NaH_2_PO_4_, 120 mM NaCl, 6 mM KCl, 1 mM CaCl_2_, 1.2 mM MgSO_4_, 12.5 mM HEPES, pH 7.4) containing 5 mM glucose and 3.5% fatty acid free BSA (Sigma) that was supplemented with or without 10 nM insulin and/or CL316,243 (1 nM for 3T3-L1 adipocytes or 0.25 nM for brown adipocytes). The dose of CL316,243 was optimized for maximal fold change of CL316,243 vs CL316,243 + insulin in control adipocytes. Glycerol release into the medium was determined after 1 h using the free glycerol reagent (Sigma) and normalized to protein content (determined by BCA) of cells lysed in PBS containing 2% SDS with phosphatase and protease inhibitors that were subsequently subjected to immunoblotting.

[^3^H]-2-deoxyglucose ([^3^H]-2DG) uptake assay was performed in 3T3-L1 and brown adipocytes grown in 24-well plates as previously described with 2–3 technical replicates per data point [3]. Briefly, cells were serum starved for 2 h in DMEM containing 0.2% BSA, followed by replacement of medium with KRP buffer containing 0.2% BSA with or without indicated doses of insulin for 20 min. Glucose uptake was initiated during the last 5 min by addition of 0.25 μCi [^3^H]-2DG (Perkin Elmer) per well in the presence of 50 μM unlabeled 2DG. 25 μM cytochalasin B was used to control for non-specific glucose uptake in separate wells. Cells were lysed with 1% Triton X-100 in PBS and assessed for radioactivity by scintillation counting and protein content for normalization.

### 2.4 Immunoblotting

For assessment of signaling, cells from lipolysis experiments were used and lysed in indicated lysis buffers at the completion of the lipolysis assay with indicated treatments (2 h basaling, followed by 1 h stimulation with or without insulin and/or CL316,243). For other immunoblots, cells or tissues were lysed in 2% SDS lysis buffer supplemented with protease and phosphatase inhibitors and protein concentration was determined by BCA assay. Samples, prepared in sample buffer containing TCEP, and Novex pre-stained molecular weight markers (Thermo Fisher Scientific) were subjected to SDS-PAGE and gels were transferred to PVDF membranes. Membranes were subjected to immunoblotting with primary and either infrared dye 700- or 800-conjugated secondary antibodies or horseradish peroxidase-labelled secondary antibodies, followed by detection with either Odyssey CLx (Li-Cor) or using ECL (Thermo Fisher Scientific or Millipore) on the ChemiDoc (Bio-Rad), respectively. Densitometry analysis was performed using ImageStudioLite (Li-Cor) and band intensities normalized to loading control (tubulin or 14-3-3).

### 2.5 TAMRA-FP serine hydrolase activity probe

3T3-L1 adipocytes were lysed in NP40 buffer (1% NP40, 137 mM NaCl, 10% glycerol, 25 mM Tris, pH 7.4, supplemented with phosphatase and protease inhibitors) and lysates were subjected to immunoprecipitation with IgG control or antibodies against HSL or ABHD15. Eluates and lysates were incubated with 2 μM ActivX TAMRA-FP Serine Hydrolase Probe (Thermo Fisher Scientific) and subsequently subjected to SDS-PAGE. In-gel fluorescent scanning with the Typhoon FLA 9500 (GE Healthcare) was used to detect TAMRA-FP labelled proteins and Odyssey CLx was used to detect the Novex pre-stained molecular weight marker. The same gel was subsequently transferred to PVDF, followed by immunoblotting with antibodies against HSL and ABHD15.

### 2.6 ABHD15 KO mice

ABHD15^−/−^ mice were produced by the Mouse Engineering at Garvan/ABR Facility (Moss Vale and Sydney, Australia) using CRISPR/Cas9 gene targeting in C57BL/6J mouse embryos following established molecular and animal husbandry techniques [18]. The single guide RNA (sgRNA) employed targeted within Exon 1 of *ABHD15* (CATTTGCAAGCCGTCGGCGCTGGprotospacer-associated motif = PAM underlined). A solution consisting of sgRNA (15 ng/μl) and full length, polyadenylated *S. pyogenes* Cas9 mRNA (30 ng/μl) was prepared and microinjected into the nucleus and cytoplasm of C57BL/6J zygotes. Microinjected embryos were cultured overnight and those that underwent cleavage were introduced into pseudo-pregnant foster mothers. Pups were screened by PCR across the target site and Sanger sequencing of PCR products to detect modifications to *ABHD15.* Independent founders carrying 1 bp, 65 bp and 100 bp frame-shift deletions within Exon 1 (around Pro67) were identified, crossed with wild-type C57BL/6J mice and progeny inter-crossed to produce the three ABHD15^−/−^ lines.

The following primers were used for genotyping: ABHD15_1bp-del_F1: 5′-AGGCTGCAGCCTCATTTGCAA-3′, ABHD15_1bp-del_F2: 5′-CCTCATTTGCAAGCCGTCGG-3′, ABHD15_1bp-del_R: 5′-CGAGCGTCGCAGAGCGCGCAGC-3′, ABHD15_65bp-del_F: 5′-GTTCAGCGACCGGCGCGAAG-3′, ABHD15_65bp-del_R: 5′-TGGGGTCCCGACAGCCAGGAGC-3′, ABHD15_100bp-del_F: 5′-GCTCTAGCGCTGCTACTGGC-3′; ABHD15_100bp-del_R: 5′-ATTGGGGATTACCAACAGCACT-3′.

All animal experiments were carried out in accordance with the NHMRC (Australia) guidelines for animal research and were approved by the University of Sydney Animal Ethics Committee. ABHD15^+/−^ breeding pairs were used to generate all male ABHD15^−/−^ and wild type littermates used for experiments in this study (aged 9–23 wks). Mice were group-housed on a 12 h light/dark cycle with free access to food and water.

### 2.7 Metabolic measurements in mice

Body composition of mice (10–12 wks old) was determined using Echo-MRI. Glucose tolerance test (GTT) and insulin tolerance test (ITT) were performed on male mice after a 6-h fast (GTT, 10–15 wks old) or an overnight fast (ITT, 12–22 wks old) via intraperitoneal injection of a bolus of glucose (2 g/kg lean mass) or insulin (1 U/kg lean mass). For insulin injections the tail was snipped 10 min prior to the start of the experiment. At indicated time points, blood glucose and insulin were measured by sampling blood from the tail tip using an Accu-Check II glucometer (Roche Diagnostics) or an insulin ELISA kit (Crystal Chem), respectively. Plasma was obtained by collecting blood in EDTA-coated tubes (Sarstedt), followed by centrifugation at 2000×*g* for 10 min at 4 °C. Fed measurements were taken at 7 am. Plasma free fatty acids were measured using the NEFA C kit (Wako). Plasma measurements before and after insulin injection used the 0 and 10 min plasma samples obtained during the ITT. Mice were sacrificed by cervical dislocation and tissues isolated, snap frozen in liquid N_2_ and powdered under liquid N_2_. Islet isolation and glucose stimulated insulin secretion assay were performed as previously described [19]. Triglycerides were extracted from ∼30 mg tissue using 2:1 chloroform:methanol as previously described [20]. Liquid was evaporated using a GeneVac concentrator and lipids were resuspended in isopropanol. Triglycerides were measured using a triglyceride kit (Thermo Fisher Scientific) and normalized to tissue weight.

### 2.8 CL316,243 injection into mice

Mice (16–20 wks old) were intraperitoneally injected with CL316,243 (1 mg/kg lean mass) at ∼5 pm and blood was collected using EDTA-coated tubes before and 10 min after injection for isolation of plasma for fatty acid measurement (tail was snipped 10 min before the start of the experiment). Mice were sacrificed ∼16 h later by cervical dislocation and tissues removed, photographed, and frozen in liquid N_2_ for further analysis.

### 2.9 Assays in fat explants

Lipolysis and glucose uptake assays were performed in fat explants, as this system allows robust measurements with technical replicates in individual mice. Male mice (10–18 wks old) were sacrificed and epididymal visWAT was removed and chopped up into small ∼1 mm^2^ pieces to generate fat explants that were basaled for 2 h in DMEM containing 2% BSA. Lipolysis assay (glycerol release) and [^3^H]-2DG uptake were performed using 2–3 technical replicates per data point as previously described [3] with minor modifications. For [^3^H]-2DG uptake, explants were incubated in KRP containing 2% BSA and [^3^H]-2DG uptake was assessed during the last 5 min of the 20 min incubation with 0, 0.5 nM and 10 nM insulin and [^14^C]-mannitol was used to correct for extracellular [^3^H]-2DG. For lipolysis assays, explants were incubated in KRP containing 3.5% fatty acid-free BSA and 5 mM glucose, and glycerol release into the medium was measured after 1 h of treatment with or without 0.1 nM CL316,243 and/or 10 nM insulin. Fat explants were lysed in radioimmunoprecipitation assay (RIPA) buffer containing protease and phosphatase inhibitors to determine protein content by BCA assay for normalization and for subsequent immunoblotting.

### 2.10 Adipocyte isolation from WAT

Male C57Bl/6J mice for adipocyte proteomics were purchased from ABR (Australian BioResources) and fed chow diet (13% calories from fat, 65% carbohydrate, 22% protein) or Western diet (WD, 47% fat [7:1 lard-to-safflower oil ratio], 32% carbohydrate, 21% protein) for 9 mo. After 9 mo of diet feeding, mice were sacrificed and epididymal visceral white adipose tissue (visWAT) and inguinal subcutaneous WAT (scWAT) removed. Adipocytes were isolated from visWAT and scWAT as previously described [21] with minor modifications. WAT was minced in fresh buffer (120 mM NaCl, 4.7 mM KCl, 1.18 mM KH_2_PO_4_, 1.17 mM MgSO_4_.7H_2_O, 2 mM CaCl_2_, 30 mM HEPES, 10 mM NaHCO_3_, 5 mM glucose, 1% BSA, pH 7.4) until pieces were <1 mm^2^ in size. For digestion, type I collagenase (Worthington) was added at a concentration of 0.5 mg/ml for visWAT and 1 mg/ml for scWAT, and samples placed in a shaking water bath at 100 rpm for 1 h at 37 °C. Samples were then filtered through a 250 μm or 300 μm nylon mesh (Spectrum Labs) for chow- and WD-fed mice, respectively, and washed 3 times with HES buffer (250 mM sucrose, 20 mM HEPES, 1 mM EDTA, pH 7.4). Between washes, adipocytes were left for 5 min to form a floating layer and the infranatant removed by aspiration using a Hamilton syringe.

### 2.11 Mass spectrometry - sample preparation, MS analysis and data analysis

Adipocytes were lysed and processed according to the iST protocol [22]. Briefly, cells were lysed in equal volumes of SDC lysis buffer (2% sodium deoxycholate, 200 mM Tris HCl pH 8.5) and samples were boiled at 95 °C for 5 min with mixing, then cooled on ice. Samples were spun at 21,000×*g* for 15 min at 0 °C, and the fat layer was carefully discarded. Protein quantification was performed using BCA assay. 60 μg of protein was transferred to clean tubes, diluted to equal volumes, and protein was reduced with 10 mM TCEP and alkylated with 40 mM 2-choroacetamide at 95 °C for 5 min. Trypsin (Sigma) and LysC (Wako) were added at a ratio of 1 μg enzyme to 50 μg protein, and samples digested at 37 °C overnight for 18 h with mixing (2,000 rpm). Digested peptides were desalted on SDB-RPS (styrenedivinylbenzene-reverse phase sulfonate, 3M Empore) StageTips as follows. Samples were diluted 50% with 99% EA (ethyl acetate)/1% TFA (trifluoracetic acid), vortexed thoroughly, and loaded onto StageTips packed with 2x disks SDB-RPS material. StageTips were washed 1x with 100 μl 99% EA/1% TFA, and 2x with 100 μl 0.2% TFA, then eluted with 5% ammonia/80% ACN (acetonitrile) and dried in a vacuum concentrator (Eppendorf). Peptides were subsequently separated into three fractions using StageTip-based SCX (strong cation exchange) fractionation [23]. Briefly, ∼30 μg of peptides were resuspended in 1% TFA and loaded onto StageTips packed with 6x disks of SCX material (3M Empore). Peptides were eluted and collected separately with increasing concentrations of ammonium acetate (150 mM and 300 mM) in 20% ACN, followed by 5% ammonia/80% ACN. Collected peptide fractions were dried in a vacuum concentrator (Eppendorf), and resuspended in MS loading buffer (2% ACN/0.3% TFA). Peptides were analyzed by mass spectrometry using a Dionex Ultimate 3000 UHPLC coupled to a Q Exactive Plus benchtop Orbitrap Mass Spectrometer (Thermo Fisher Scientific). Peptides were loaded onto an in-house packed 75 μm ID x 50 cm column packed with 1.9 μm C18 material (Dr Maisch, ReproSil Pur C18-AQ), and separated with a gradient of 5–30% ACN containing 0.1% FA over 95 min at 300 nl/min, and column temperature was maintained at 60 °C with a column oven (Sonation). MS1 scans were acquired from 300 to 1,650 *m*/*z* (35,000 resolution, 3e6 fill target, 20 ms maximum fill time), followed by MS/MS data-dependent acquisition of the top 15 ions using high-energy dissociation (HCD), with MS2 fragment ions read out in the Orbitrap (17,500 resolution, 1e5 AGC, 25 ms maximum fill time, 25 NCE, 1.4 *m*/*z* isolation width).

Raw data were processed using MaxQuant (version 1.5.9.1) [24] searched against a UniProt mouse database (January 2016 release). Default settings were used, and match between runs was switched on to facilitate the transfer of MS/MS identifications between equivalent and adjacent fraction measurements. The data were then filtered to remove contaminants and proteins that were not quantified in any sample, and protein intensities were transformed to the log2 scale using the R statistical software.

### 2.12 Statistical analysis

Data are presented as mean ± SEM with individual data points shown, unless otherwise indicated. For glucose uptake and lipolysis experiments, each data point is an independent experiment and represents the average of 2–3 technical replicates. Statistical analyses were performed in GraphPad Prism or software R using two-tailed t-test (if not otherwise indicated) or one-way or two-way analysis of variance (ANOVA) with post-hoc analysis using Tukey's or Sidak's multiple comparison tests. Significance was set at p < 0.05 and p-values are indicated.

## 3 Results

### 3.1 Deletion of ABHD15 resulted in reduced PDE3B and impaired insulin regulation of lipolysis

**Figure 1:**
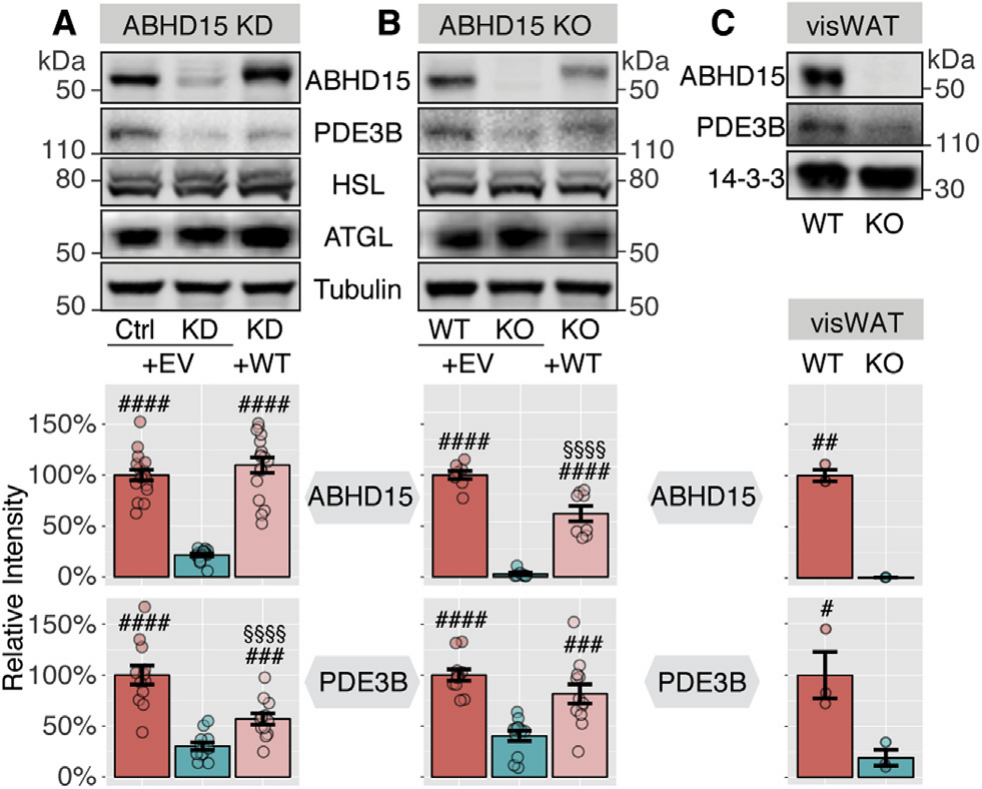
**Loss of ABHD15 resulted in reduced PDE3B.** Tissue or cells were lysed and immunoblotted with indicated antibodies. Loss of ABHD15 models include (**A**) 3T3-L1 adipocytes infected with shRNA against ABHD15 (KD) or control (Ctrl), (**B**) wild type (WT) and ABHD15^−/−^ (KO) brown adipocytes, and (**C**) visWAT from wild type and ABHD15^−/−^ mice. ABHD15 was re-expressed (+WT) in knockdown (**A**) or knockout adipocytes (**B**) with control cells infected with empty vector (+EV). Representative images are shown. ABHD15 and PDE3B protein levels were quantified. Data are mean ± SEM with individual data points shown (**A**: n = 12–17; **B**: 8–12; **C**: n = 3). # different from KD/KO, #p < 0.05, ##p < 0.01, ###p < 0.001, ####p < 0.0001; §§§§p < 0.0001 Ctrl/WT vs. KD + WT/KO + WT; statistical significance was determined using one-way ANOVA with Tukey's multiple comparison test (**A**, **B**) or two-tailed t-test (**C**).

ABHD15 was first identified as an insulin-regulated phosphoprotein that interacts with PDE3B by Lienhard and colleagues [8], [10]. Our laboratory subsequently identified this protein as one of the most highly insulin-responsive phosphoproteins in an unbiased phosphoproteomics screen in adipocytes [9]. Similar to PDE3B, the expression of ABHD15 is induced during 3T3-L1 adipocyte differentiation and ABHD15 knock down in 3T3-L1 adipocytes resulted in loss of the PDE3B protein [8]. To further pursue the role of ABHD15 in lipolysis, we generated several ABHD15 knockdown and knockout adipocyte models: ABHD15 knockdown was achieved in 3T3-L1 adipocytes, using ABHD15 shRNA [8] expressed via retrovirus. ABHD15^−/−^ adipocytes and fat explants both originated from global ABHD15^−/−^ mice that were created using CrispR technology. Fat explants were prepared using visceral white adipose tissue (visWAT) fat pads, while an ABHD15^−/−^ brown adipocyte cell line was obtained following isolation of brown preadipocytes from ABHD15^−/−^ newborn mice that were subsequently immortalized. Appropriate controls were used for each adipocyte model (shRNA against luciferase, brown adipocytes from wild type newborn littermate, or visWAT from wild type littermates). ABHD15 protein was reduced by ≥80% in all three ABHD15 deletion models compared to their controls (Figure 1). PDE3B protein levels were reduced by ≥60% in ABHD15 knockdown/knockout cells and this effect was rescued by ABHD15 re-expression (Figure 1). ABHD15 deletion did not affect adipocyte differentiation as indicated by the unchanged levels of adipocyte-specific proteins, such as HSL and ATGL (Figure 1).

**Figure 2:**
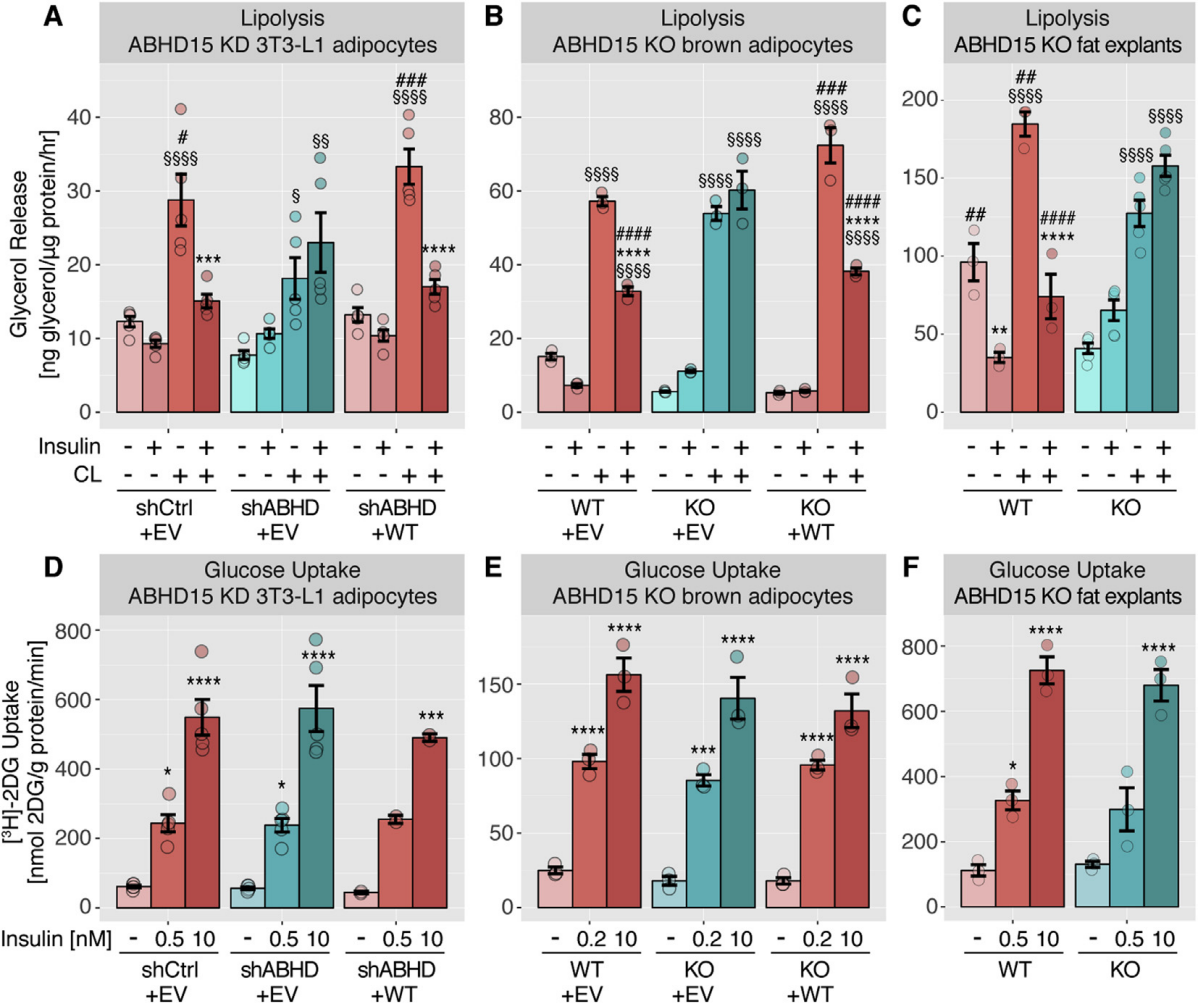
**Loss of ABHD15 resulted in impaired insulin regulation of lipolysis with no defect in glucose uptake.** Lipolysis (**A-C**) and glucose uptake (**D-F**) was assessed either in 3T3-L1 adipocytes infected with shRNA against ABHD15 (shABHD) or control (shCtrl, **A**, **D**), or in wild type (WT) and ABHD15^−/−^ (KO) brown adipocytes (**B**, **E**), or in visWAT explants from wild type and ABHD15^−/−^ mice (**C**, **F**). ABHD15 was re-expressed (+WT) in knockdown (**A**, **D**) or knockout adipocytes (**B**, **E**) with control cells infected with empty vector (+EV). Glycerol release (**A-C**) or [^3^H]-2-deoxyglucose ([^3^H]-2DG) uptake (**D-F**) was assessed in adipocytes or fat explants in response to indicated treatments (CL, CL316,243). Data are mean ± SEM with individual data points shown (n = 3–5, except **D**: KD + WT (n = 2, mean ± SD)); * different from without insulin, *p < 0.05, **p < 0.01, ***p < 0.001, ****p < 0.0001; § different from without CL, §p < 0.05, §§p < 0.01, §§§§p < 0.0001; # different from KD/KO, #p < 0.05, ##p < 0.01, ###p < 0.001, ####p < 0.0001; statistical significance was determined using two-way ANOVA with Tukey's multiple comparison test.

We next assessed the role of ABHD15 in lipolysis. ABHD15 deletion and control cells were incubated in the presence or absence of the β3-adrenergic receptor agonist CL316,243 and/or insulin and glycerol release was assessed as a measure of lipolysis. Across all three loss of ABHD15 models, CL316,243 significantly stimulated lipolysis in both control cells as well as ABHD15 deletion cells (Figure 2A–C). Insulin suppressed CL316,243-induced lipolysis in control cells, but this suppression was abolished in all three ABHD15 deletion cell models, indicating that ABHD15 is essential for insulin regulation of lipolysis. Re-expression of wild type ABHD15 in knockdown and knockout adipocytes rescued insulin regulation of lipolysis, indicating that the impaired ability of insulin to regulate lipolysis in ABHD15 deletion adipocytes is due to loss of AHBD15 and not off-target effects (Figure 2A–B). In fat explants, a reduction in basal and CL316,243-stimulated lipolysis was also observed in ABHD15^−/−^ fat explants compared to wild type fat explants. This defect was particularly evident in fat tissue explants and less marked or not present in the adipocyte cell line models, indicating that fat explants may also be influenced by systemic, cell non-autonomous factors. The impaired insulin regulation of lipolysis was not due to a general defect in insulin action because insulin-stimulated glucose uptake, as determined by [^3^H]-2-deoxyglucose uptake (Figure 2D–F), and Akt phosphorylation (see 3.3, Figure 4) was unaffected by loss of ABHD15 across all models.

**Figure 3:**
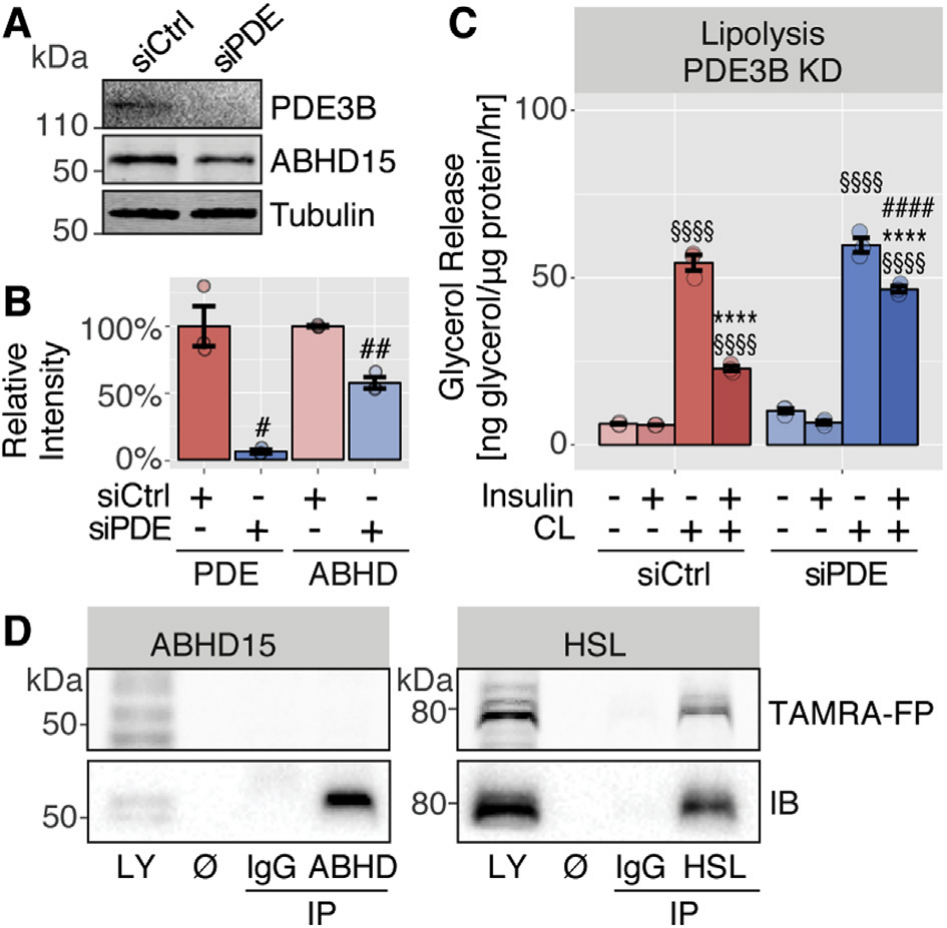
**ABHD15 affected lipolysis in a PDE3B-independent manner and showed no catalytic activity. A**, 3T3-L1 adipocytes were transfected with scrambled control (siCtrl) or PDE3B siRNA (siPDE) and cell lysates immunoblotted with indicated antibodies. Representative immunoblots are shown. **B**, Data in A were quantified (n = 3). **C**, Lipolysis was assessed in siCtrl and siPDE adipocytes by measuring glycerol release in response to indicated treatments (CL, CL316,243) (n = 3). **D**, 3T3-L1 adipocyte lysate was subjected to immunoprecipitation (IP) with IgG, ABHD15 or HSL antibodies and both lysate (LY) and IPs were incubated with hydrolase activity probe TAMRA-FP and subjected to SDS-PAGE. TAMRA-FP labelled proteins were visualized by in-gel fluorescent scanning, and ABHD15 and HSL were detected by immunoblotting. Representative images (TAMRA-FP scan and immunoblots, IB) of the molecular weight of ABHD15 and HSL are shown. Data are mean ± SEM with individual data points shown; **** different from without insulin p < 0.0001; §§§§ different from without CL p < 0.0001; # different from corresponding siCtrl, #p < 0.05, ###p < 0.001, ####p < 0.0001; statistical significance was determined using two-tailed t-test (**B**) or two-way ANOVA with Tukey's multiple comparison test (**C**).

**Figure 4:**
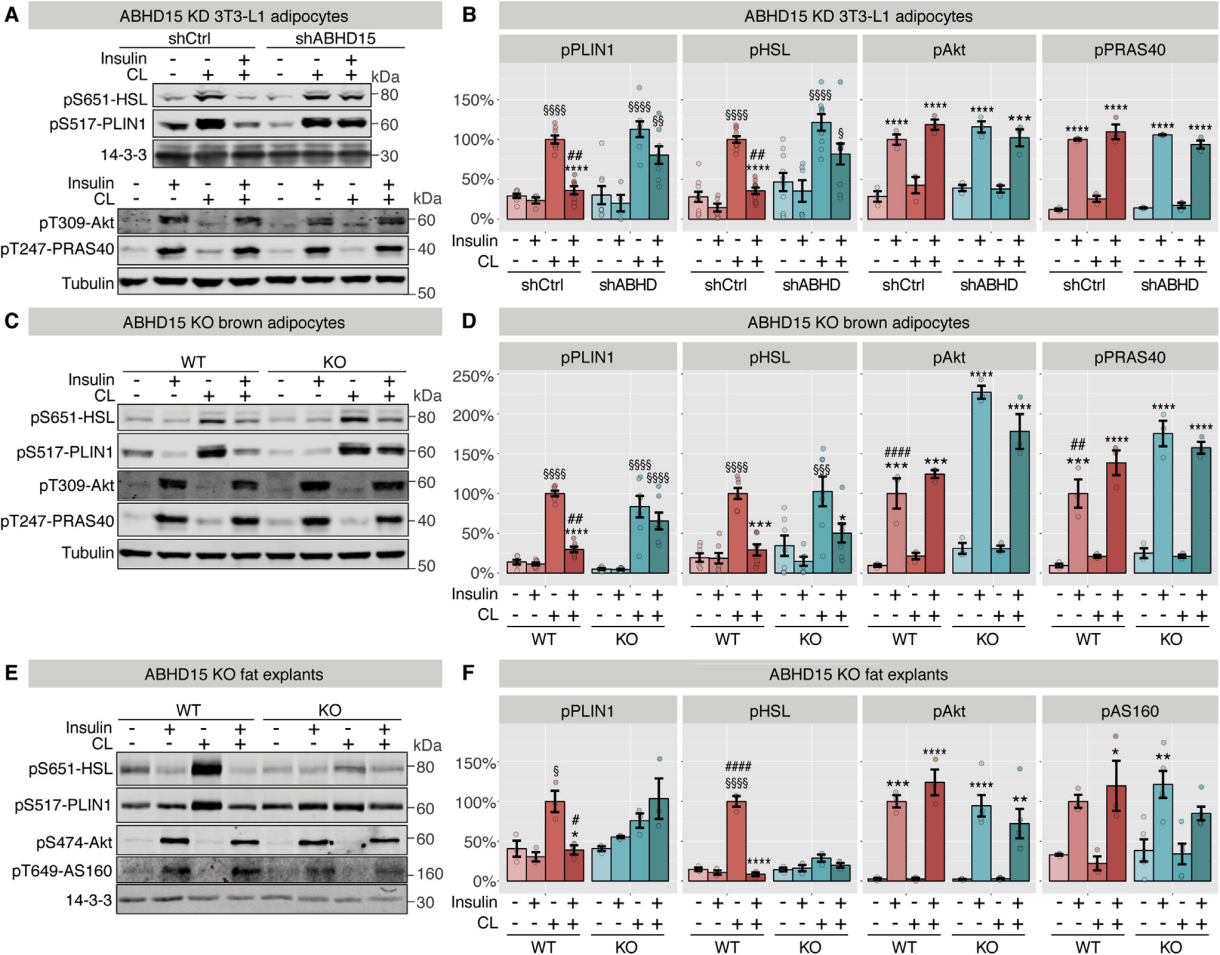
**Loss of ABHD15 affected regulation of PLIN1 phosphorylation.** Signaling was assessed after completion of lipolysis assays either in 3T3-L1 adipocytes infected with shRNA against ABHD15 (shABHD) or control (shCtrl, **A-B**), or in wild type (WT) and ABHD15^−/−^ (KO) brown adipocytes (**C-D**), or in visWAT explants from wild type and ABHD15^−/−^ mice (**E-F**). Cells lysates were subjected to SDS-PAGE and immunoblotting with indicated antibodies. Representative immunoblots and quantification are shown (**B** (n = 3–10), **D** (n = 3–7), **F** (n = 3–5)). Data are mean ± SEM with individual data points shown; * different from without insulin, *p < 0.05, **p < 0.01, ***p < 0.001, ****p < 0.0001; § different from without CL, §p < 0.05, §§p < 0.01, §§§p < 0.001, §§§§p < 0.0001; # different from KD/KO, #p < 0.05, ##p < 0.01, ####p < 0.0001; statistical significance was determined using two-way ANOVA with Tukey's multiple comparison test.

### 3.2 ABHD15 affected lipolysis in a PDE3B-independent manner and showed no catalytic activity

As loss of ABHD15 also led to a concomitant reduction in PDE3B levels and deletion of PDE3B resulted in impaired insulin regulation of lipolysis, it is possible that the observed lipolysis defect in ABHD15 deletion cells was solely due to loss of PDE3B. Therefore, we next assessed lipolysis in PDE3B knockdown adipocytes. PDE3B was efficiently reduced by 94% using siRNA targeting PDE3B (Figure 3A–B). Interestingly, ABHD15 protein levels were also reduced in this model suggesting a reciprocal relationship between these two proteins, likely involving the formation of a stable dimeric complex. However, ABHD15 was only reduced by 42%, indicating that it may be partially stable in the absence of PDE3B (Figure 3A–B). As previously reported in PDE3B knockout adipocytes, insulin-mediated suppression of CL316,243-induced lipolysis was impaired in PDE3B knockdown cells (Figure 3C). However, in contrast to cells lacking ABHD15, where insulin regulation of lipolysis was completely blocked (Figure 2), in PDE3B knockdown cells lipolysis suppression was only partial, with a small but significant insulin-mediated reduction of CL316,243-induced lipolysis (Figure 3C). These data indicate that the lipolysis defects observed in the absence of ABHD15 were not solely due to the loss of PDE3B.

One of the PDE3B-independent actions of ABHD15 might involve its hydrolase activity. Catalytically active α/β-hydrolases contain a serine within their catalytic triad, however, this is not present in ABHD15, suggesting that ABHD15 does not display hydrolase activity [7]. Nevertheless, we next investigated whether ABHD15 harbours catalytic hydrolase activity, by testing whether ABHD15 could interact with the fluorescently labelled serine hydrolase activity probe TAMRA-FP [7]. 3T3-L1 adipocyte lysate was subjected to immunoprecipitation with antibodies against ABHD15, HSL or IgG control and eluates as well as lysate were incubated with TAMRA-FP. Lysate and immunoprecipitated proteins were subjected to SDS-PAGE, followed by in-gel fluorescent scanning to detect TAMRA-FP labelled proteins and subsequent immunoblotting with ABHD15 or HSL antibodies. While the active α/β-hydrolase HSL was labeled by the activity probe, this was not the case for ABHD15 (Figure 3D), indicating that ABHD15 has no hydrolase activity. This is consistent with the absence of a catalytic serine residue. In agreement with this, a hydrolase activity screen in adipocytes failed to identify ABHD15 [25].

### 3.3 Loss of ABHD15 affected regulation of PLIN1 phosphorylation

To determine whether the lipolysis defect observed in ABHD15 deletion adipocytes was associated with defective PKA signaling, insulin and β-adrenergic receptor agonist-mediated protein phosphorylation was assessed. The same adipocytes and fat explants that were subjected to lipolysis experiments were subsequently lysed and subjected to SDS-PAGE and immunoblotting with antibodies against signaling molecules comprising the insulin and β-adrenergic receptor pathways, including phospho-specific antibodies against the PKA substrates PLIN1 and HSL, as well as Akt and the Akt substrates AS160 and PRAS40. Consistent with normal insulin-stimulated glucose uptake in ABHD15 deletion cells (Figure 2D–F), no defect was observed in insulin signaling in ABHD15 knockdown/knockout cells, as indicated by increased phosphorylation of Akt and the Akt substrates AS160 and PRAS40 (Figure 4). We next assessed PKA signaling, focusing on the PKA substrates that are specifically involved in lipolysis, PLIN1 and HSL. Phosphorylation of PLIN1 is essential for the recruitment and activation of ATGL, the first lipase in triglyceride hydrolysis, while phosphorylation of HSL activates the second lipase in lipolysis; hence, phosphorylation of both these PKA substrates is crucial for lipolysis regulation [1]. As expected, in all control cells, PKA-mediated phosphorylation of PLIN1 and HSL was elevated in response to the β-adrenergic receptor agonist CL316,243 and this response was suppressed by insulin (Figure 4). In ABHD15 deletion cells, while CL316,243 triggered a similar increase in PLIN1 phosphorylation to that seen in control cells, its insulin-dependent suppression was impaired across all models. HSL phosphorylation showed considerable variability between the three model systems in the absence of ABHD15. While HSL phosphorylation was unaffected in ABHD15^−/−^ adipocytes (Figure 4C–D), in ABHD15 knockdown adipocytes insulin suppression of CL316,243-induced phospho-HSL was impaired (Figure 4A–B). The response in ABHD15^−/−^ fat explants was even more divergent as no increase in phospho-HSL was observed with CL316,243 stimulation (Figure 4E–F). This lack of responsiveness to CL316,243 might either be due to whole body systemic factors that are maintained throughout the *ex vivo* experiment duration, or this may reflect changes in the timing of PKA activity as it has been shown that different phosphorylation sites in HSL showed distinct temporal patterns in response to β-adrenergic receptor agonists [26]. In summary, the common defect across all models converged on the PLIN1 phosphorylation that was not suppressed by insulin in all three loss of ABHD15 models. Notably, the pattern of phospho-PLIN1 was remarkably similar to the lipolysis response that was observed in each of the three models (Figure 2, Figure 4).

### 3.4 Elevated plasma fatty acids upon fasting and in response to insulin in ABHD15^−/−^ mice

**Figure 5:**
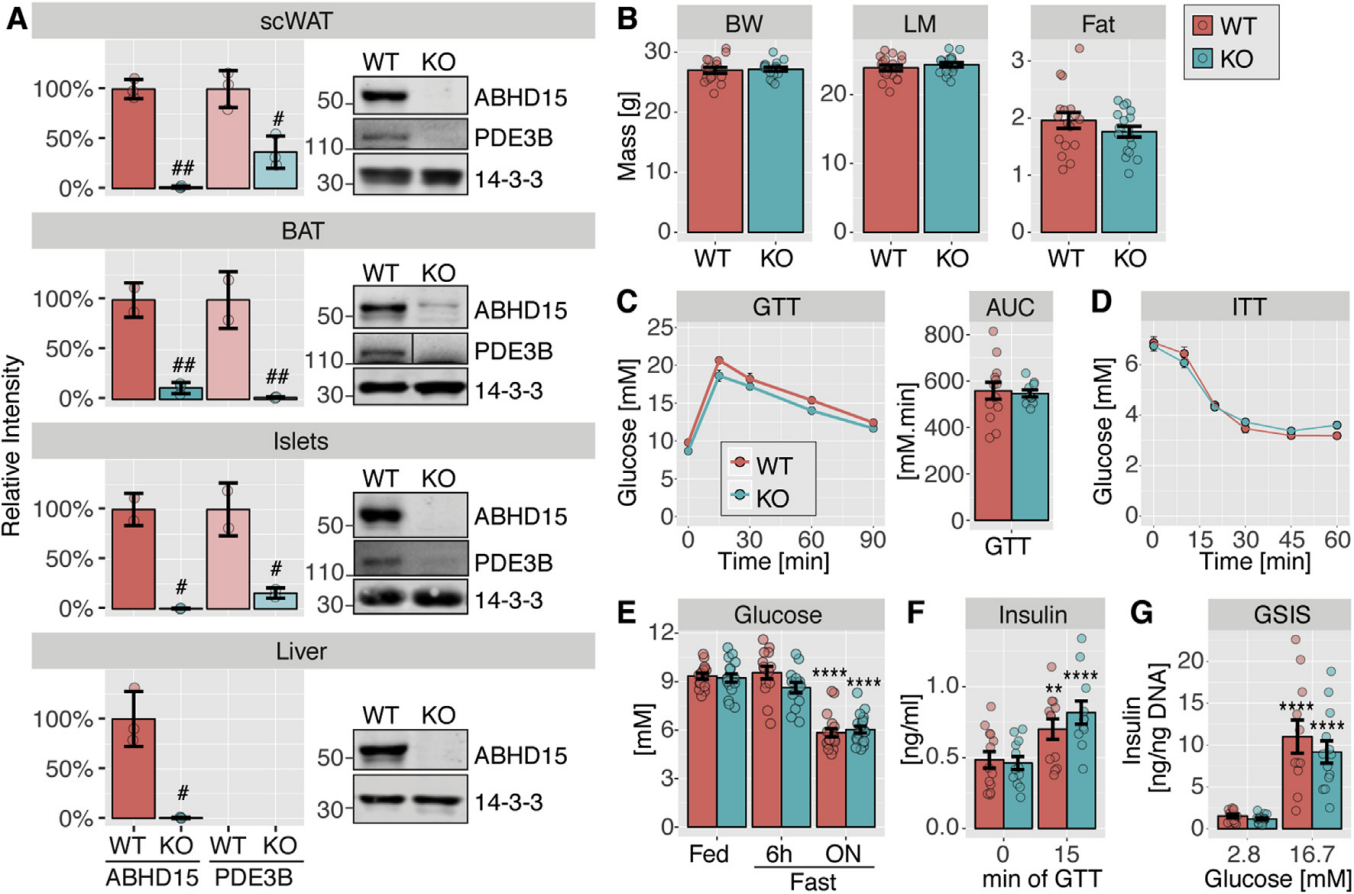
**ABHD15**^**−/−**^**mice showed no defects in glucose metabolism or insulin secretion. A-G**, ABHD15^−/−^ (KO) mice and wild type (WT) littermates were subjected to phenotypic characterisation. **A**, Mice were culled, tissues removed and subjected to immunoblotting with indicated antibodies, followed by quantification (scWAT, subcutaneous white adipose tissue; BAT, brown adipose tissue; n = 3, except BAT, islets: n = 2). **B**, Body weight (BW), lean mass (LM) and fat mass was determined (n = 16–17). **C**, Mice were subjected to glucose tolerance test (GTT) and area under the curve (AUC) was calculated (n = 10–13). **D**, Mice were subjected to insulin tolerance test (ITT) (n = 18–19). **E**, Blood glucose was measured under fed, 6-h fasted and overnight (ON) fasted conditions (n = 14–19). **F**, Blood insulin was measured at 0 and 15 min during the GTT (n = 11–13). **G**, Mice were culled, and pancreatic islets were isolated for assessment of glucose-stimulated insulin secretion (GSIS, n = 11–14). Data are mean ± SD (**A**) or mean ± SEM (**B-G**) with individual data points shown (except for ITT and GTT); * different from other conditions of the same genotype, *p < 0.05, ***p < 0.001, ****p < 0.0001; # different from WT, ##p < 0.01, ###p < 0.001, ####p < 0.0001; statistical significance was determined using two-tailed t-test (**A-C**) or two-way ANOVA with Tukey's or Sidak's multiple comparison test (**E-G**).

Next, we assessed the effects of ABHD15 deletion on whole body metabolism. Since the genetic deletion of ABHD15 was global in our mouse model, and ABHD15 expression was reported in other insulin-responsive tissues such as pancreas and liver in addition to adipose tissue [8], ABHD15 and PDE3B protein expression was determined in all these tissues. Similar to visWAT (Figure 1), a ≥90% reduction in ABHD15 protein was observed in subcutaneous WAT (scWAT), brown adipose tissue, as well as in isolated pancreatic islets and liver (Figure 5A). ABHD15 protein levels could not be determined in pancreas due to a strong non-specific band at a similar molecular weight (data not shown); hence, isolated pancreatic islets were used. As expected, PDE3B protein levels were also reduced ≥60% in scWAT, brown adipose tissue and pancreatic islets from ABHD15^−/−^ mice. PDE3B was not detectable in wild type liver. We next assessed whether lack of ABHD15 affected body composition or whole-body glucose metabolism, but no significant differences were observed in body weight, fat mass, or lean mass between ABHD15^−/−^ mice and their wild type littermates (Figure 5B). ABHD15^−/−^ mice showed normal glucose metabolism as assessed by analysis of blood glucose levels during an intraperitoneal glucose tolerance test (GTT), an insulin tolerance test (ITT) and in the fed and fasted (6-h or overnight) states (Figure 5C–E). In contrast to the PDE3B^−/−^ mice [4] we did not observe elevated insulin secretion in ABHD15^−/−^ mice. Blood insulin levels were normal during the GTT with a significant elevation after 15 min (Figure 5F), and similarly no difference was observed in insulin secretion in isolated islets, with islets from both genotypes displaying a significant glucose-dependent increase (Figure 5G).

**Figure 6:**
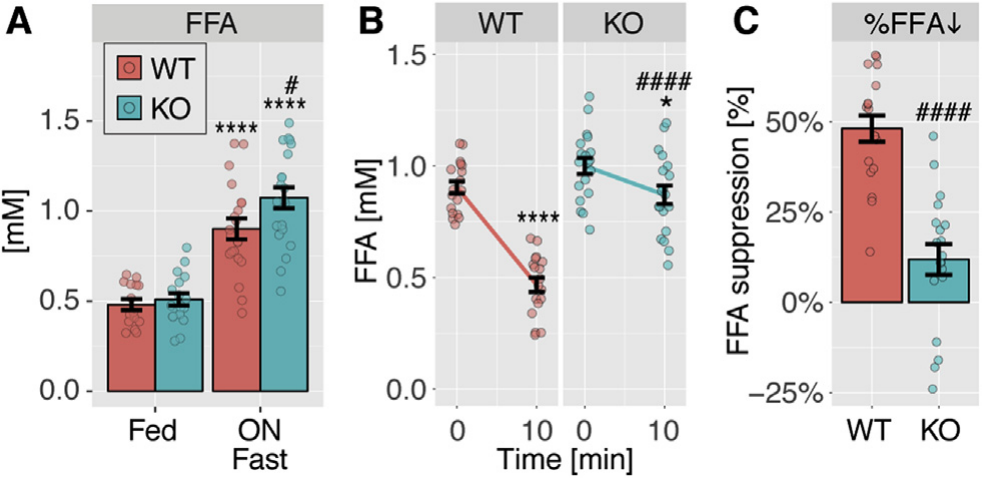
**Elevated plasma fatty acids upon fasting and in response to insulin in ABHD15**^**−/−**^**mice. A**, Free fatty acids (FFA) were measured in plasma from ABHD15^−/−^ (KO) and wild type (WT) mice under fed and overnight (ON) fasted conditions (n = 15–21). **B**, Plasma fatty acids were measured before and 10 min after insulin injection in ABHD15^−/−^ and wild type mice (n = 18–19). **C**, Percentage of plasma fatty acid suppression (%FFA) was calculated for each individual mouse of data in **B**. Data are mean ± SEM with individual data points shown; * different from fed (A) or 0 min (B), *p < 0.05, ****p < 0.0001; # different from WT, #p < 0.05, ####p < 0.0001; statistical significance was determined using two-way ANOVA with Sidak's multiple comparison test (**A-B**) or two-tailed t-test (**C**).

In view of the defective lipolysis observed in ABHD15^−/−^ WAT *ex vivo* (Figure 2C), we next focused on fatty acid metabolism in ABHD15^−/−^ mice. There was no difference between genotypes in plasma fatty acids in fed conditions, but fatty acids were significantly elevated after an overnight fast in ABHD15^−/−^ mice compared to wild type littermates (Figure 6A). We also assessed insulin-mediated suppression of adipose tissue lipolysis *in vivo* by measuring plasma fatty acids before and after intraperitoneal insulin injection. In wild type mice, plasma fatty acids were maximally suppressed at 10 min after insulin injection (data not shown). Therefore, plasma fatty acids were assessed before and 10 min after insulin injection. Insulin significantly suppressed plasma fatty acids by almost 50% in wild type mice (Figure 6B–C), and this effect was significantly impaired in ABHD15^−/−^ mice (Figure 6B–C), in agreement with previous reports [27]. Notably, both genotypes showed a significant reduction in blood glucose in response to insulin (Figure 5D). Consistent with the lipolysis defect observed in fat explants *ex vivo*, ABHD15^−/−^ mice showed significantly impaired lipolysis suppression in response to insulin *in vivo*. In summary, plasma fatty acids were elevated in ABHD15^−/−^ mice compared to wild type littermates after overnight fasting and in response to insulin.

### 3.5 Loss of ABHD15 resulted in elevated liver lipids in response to β-adrenergic receptor agonist in mice

**Figure 7:**
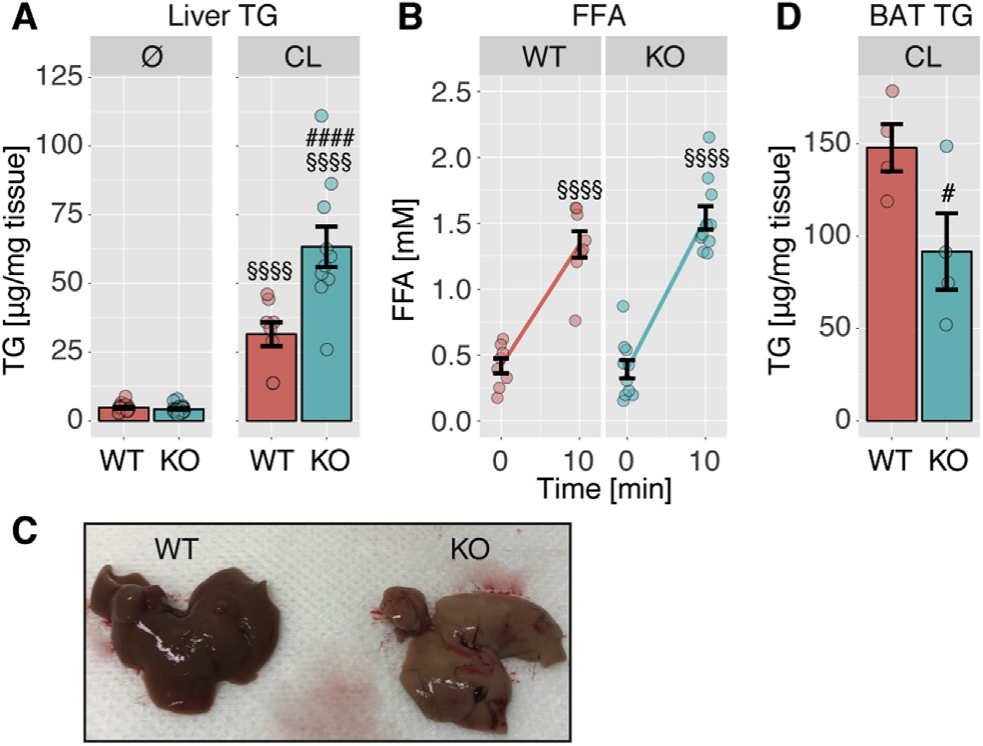
**Loss of ABHD15 resulted in elevated liver lipid in response to β-adrenergic receptor agonist in mice. A**, Liver triglycerides (TG) were measured in untreated (Ø) wild type and ABHD15^−/−^ (KO) mice (n = 20–22) or ∼16 h after CL316,243 (CL) injection (n = 8–10). **B**, Plasma fatty acids were measured before and 10 min after CL316,243 injection (n = 8–10). **C**, Representative photograph of livers from wild type and ABHD15^−/−^ mice ∼16 h after CL316,243 injection is shown. **D**, Brown adipose tissue (BAT) triglycerides were measured ∼16 h after CL316,243 injection (n = 4). Data are mean ± SEM with individual data points shown; # different from WT, #p < 0.05, ####p < 0.0001; §§§§ different from 0 min (B) or Ø (A) p < 0.0001; statistical significance was determined using two-way ANOVA with Sidak's multiple comparison test (**A-B**) or one-tailed t-test (**C**).

Impaired regulation of lipolysis has been shown to affect liver lipid accumulation. Inhibition of lipolysis using pharmacological inhibitors against ATGL or HSL resulted in reduced liver lipid among other metabolic improvements [28], [29] and it is conceivable that the failure of insulin to suppress lipolysis adequately in ABHD15^−/−^ mice might result in fatty liver and elevated liver triglycerides. However, no difference was observed in liver triglyceride levels between the genotypes (Figure 7A). The lack of an observed effect might be due to whole-body adaptations in order to ensure metabolic homeostasis; therefore, we induced adipose tissue lipolysis by injecting mice with CL316,243 towards the end of the light phase. CL316,243 resulted in a rapid (10 min) increase in plasma fatty acids in wild type and ABHD15^−/−^ littermates (Figure 7B). After the dark phase, at 16 h after CL316,243 injection, liver triglyceride content was elevated by 2-fold in ABHD15^−/−^ mice compared to wild type mice (Figure 7A,C). Brown adipose tissue was darker in ABHD15^−/−^ mice compared to wild type mice but this was not associated with a change in mitochondrial content or UCP1 levels (data not shown). However, brown adipose tissue triglyceride levels were significantly lower in ABHD15^−/−^ mice compared to wild type littermates (Figure 7D), suggesting enhanced lipolysis in ABHD15^−/−^ mice. In summary, stress-induced lipid accumulation in the liver was markedly elevated in conditions of hyperactive lipolysis as is the case in the absence of ABHD15.

### 3.6 Reduced ABHD15 and PDE3B protein levels in insulin resistant adipocytes

**Figure 8:**
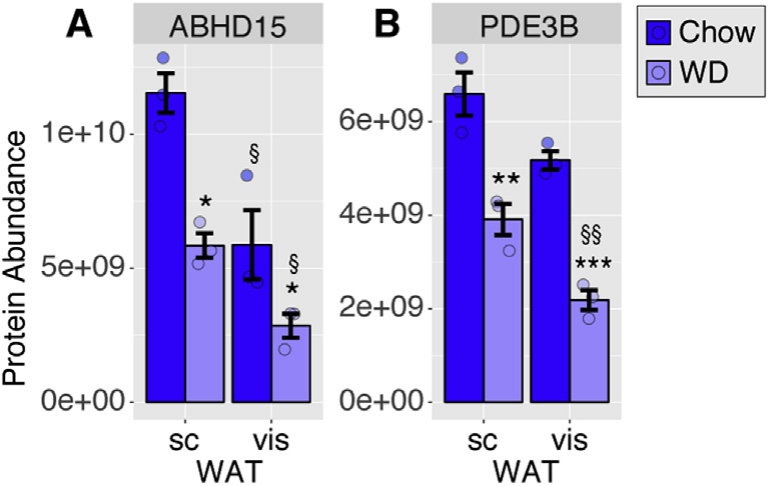
**Reduced ABHD15 and PDE3B protein levels in insulin resistant adipocytes.** Adipocytes were isolated from visWAT and scWAT from C57Bl/6J mice fed chow or Western diet (WD) for 9 mo and subjected to mass spectrometry analysis. Protein abundance is shown as average protein intensities for ABHD15 (**A**) and PDE3B (**B**). Data are mean ± SEM with individual data points shown (n = 3); * different from chow, *p < 0.05, **p < 0.01, ***p < 0.001; # different from scWAT, §p < 0.05, §§p < 0.01; statistical significance was determined using two-way ANOVA with Sidak's multiple comparison test.

Lipolysis is dysregulated in insulin resistant cells, mice, and humans [30], [31]. In view of dysregulated lipolysis in the absence of ABHD15 observed in this study, it was of interest to determine whether ABHD15 was altered in insulin resistance. C57Bl/6J mice were fed a Western diet for 9 mo and developed insulin resistance, assessed by GTT, and hyperinsulinemia compared to chow-fed mice (data not shown). VisWAT and scWAT pads were removed, followed by adipocyte isolation, which were subsequently processed for label-free mass spectrometry (MS) analysis. Data analysis revealed that ABHD15 was significantly higher in adipocytes from scWAT than those from visWAT (Figure 8A, Supplementary Table A), which is interesting as scWAT was reported to be more insulin sensitive than visWAT in suppressing lipolysis [32]. PDE3B levels showed a similar trend but this was only significant in adipocytes from Western diet fed mice (Figure 8B). Notably, ABHD15 and PDE3B protein levels were both significantly reduced in adipocytes from Western diet fed mice in both WAT depots (Figure 8). These data indicate that ABHD15 and PDE3B protein levels appear to correlate with insulin sensitivity particularly in regard to lipolysis.

## 4 Discussion

Here we show that the α/β-hydrolase domain-containing protein ABHD15 plays a major role in insulin-mediated suppression of lipolysis in adipocytes. We use several different model systems, including genetic deletion of ABHD15 in mice, to show that loss of ABHD15 is accompanied by hyperactive adipocyte lipolysis (Figure 2, Figure 6). This was associated with aberrant PLIN1 phosphorylation that was not suppressed with insulin in the absence of ABHD15 (Figure 4). The absence of ABHD15 in combination with stress led to a marked increase in liver lipid levels due to hyperactive adipocyte lipolysis (Figure 7), highlighting the important role of adipocyte lipolysis in non-alcoholic fatty liver disease (NAFLD) and other liver defects.

Initially described as a novel insulin-regulated phosphoprotein that showed increased expression during adipocyte differentiation [10], ABHD15 was subsequently shown to interact with and stabilize PDE3B [8]. Another group found a role for ABHD15 in adipogenesis [33], and more recently they reported that deletion of ABHD15 in mice resulted in a generalized defect in insulin signaling and action resulting in impaired lipolysis and glucose metabolism [27]. While our studies concur on some aspects, such as the requirement for ABHD15 in insulin mediated suppression of plasma free fatty acids, there are several notable mechanistic differences worthy of mention. Our studies show that ABHD15 has a specific role in regulating insulin-mediated suppression of lipolysis while other actions of insulin were unaffected by deletion of ABHD15. We show this in three separate and well controlled model systems spanning shRNA-mediated knockdown in 3T3-L1 adipocytes, fat explants from ABHD15^−/−^ mice and immortalized brown adipocytes from ABHD15^−/−^ mice (Figure 2A–C). In all models, there was no defect in the canonical Akt signaling pathway (Figure 4) nor in adipogenesis (Figure 1A–B), and other actions of insulin like regulated glucose uptake (Figure 2D–F) were unaffected by ABHD15 deletion. In contrast, Xia et al. [27] reported that ABHD15 deletion causes global insulin resistance. We postulate that this discrepancy is attributed to technical differences. Xia et al. [27] assessed tissue specific insulin action in liver and visWAT following oral gavage of [^3^H]-2-deoxyglucose. However, 2-deoxyglucose is not absorbed from the intestine [34] and even when administered appropriately, it is not suitable for assessment of liver glucose uptake [35]. Other discrepancies are likely due to use of age-matched control mice instead of littermates or the lower mouse numbers used by Xia et al. [27]. The importance of using littermate controls from heterozygous breeding pairs rather than age-matched controls is well recognised in metabolic research [36]. We failed to observe any significant difference in glucose or insulin tolerance *in vivo* between wild type and ABHD15^−/−^ mice (Figure 5), consistent with no defect in glucose uptake (Figure 2D–F) or insulin signaling in all three ABHD15 deletion models (Figure 4). The Akt signaling defect reported by Xia et al. [27] utilized visWAT from mice that had been injected with insulin. Hence, while we cannot exclude the possibility that under these conditions there may be a defect in Akt signaling it is unlikely due to a cell autonomous effect of ABHD15 deletion in adipocytes but rather we suspect it is a function of the study design as described above.

ABHD15^−/−^ mice displayed impaired fatty acid metabolism, consistent with Xia et al. [27]. Fasted plasma fatty acid levels were significantly higher in ABHD15^−/−^ mice than in wild type littermates (Figure 6) and insulin was unable to suppress plasma fatty acids in ABHD15^−/−^ mice *in vivo* (Figure 6). This is reminiscent of that observed in PDE3B^−/−^ mice [4].

What is the role of ABHD15 in lipolysis? One possibility is that ABHD15 simply stabilizes PDE3B as loss of ABHD15 is also accompanied by loss of PDE3B. This seems unlikely because of the disparity in the lipolysis defect observed between PDE3B knockdown (Figure 3C) and ABHD15 deletion adipocytes (Figure 2A–B). PDE3B levels were reduced by 94% and ≥60% in PDE3B knockdown and ABHD15 deletion adipocytes, respectively (Figure 1, Figure 3B), while the defect in lipolysis was greater in cells lacking ABHD15 (Figure 2, Figure 3C). This suggests that ABHD15, while stabilizing PDE3B, has an additional role to regulate lipolysis because ABHD15 levels were reduced by only 42% in PDE3B knockdown cells (Figure 3A–B). What could this role be? ABHD15 is the third member of the α/β-hydrolase family to play a role in lipolysis, the other two being HSL and CGI-58. These proteins each possess the same 3-dimensional fold comprising α-helices and β-sheets and a catalytic triad [7]. Most family members, including HSL, show hydrolase activity while some like CGI-58 and ABHD15 do not contain a conserved catalytic serine. Consistent with a lack of ABHD15 hydrolase activity, ABHD15 did not interact with the serine hydrolase activity probe (Figure 3D). However, inactive hydrolases may retain ligand binding, as is the case with CGI-58 that binds to long-chain acyl-CoAs and this regulates its interaction with PLIN1 and lipolysis [37]. Hence it is possible that ABHD15's role in lipolysis may involve ligand binding via its α/β-hydrolase domain.

Based on our data, it is clear that ABHD15 plays a role in selective PKA signaling (Figure 4). Insulin inhibits lipolysis by lowering cAMP levels leading to inhibition of PKA and reduced phosphorylation of the PKA substrates PLIN1 and HSL (Figure 4). Notably, insulin-mediated suppression of PLIN1 phosphorylation was defective across all three ABHD15 deletion models (Figure 4). Since phosphorylation of HSL was not defective (Figure 4), this suggests that ABHD15 is somehow involved in regulating the activity of a pool of PKA located on lipid droplets because HSL is likely phosphorylated by PKA in the cytosol in contrast to PLIN1, which is phosphorylated at the lipid droplet surface. PKA is known to harbor very tight spatially controlled activity due to its interaction with A-kinase anchoring proteins (AKAPs) that target a particular action of PKA to a certain subcellular location [38]. Interestingly, PKA/AKAP complexes often contain PDE proteins, thereby allowing tight control of cAMP levels and PKA activity at specific cellular locations [38], [39]. It is possible that the ABHD15/PDE3B complex is part of such a PKA/AKAP ‘signalosome’.

It has been suggested that dysregulated lipolysis in adipocytes may contribute to NAFLD and other liver diseases. Intriguingly, when ABHD15^−/−^ mice were subjected to stress using the β-adrenergic receptor agonist CL316,243 we observed markedly elevated liver triglyceride levels (Figure 7). This was likely due to hyperactivation of adipose tissue lipolysis, because this was accompanied by a substantial reduction in brown adipose tissue triglycerides (Figure 7). These data highlight that stress combined with hyperactive adipocyte lipolysis represents a major risk factor for NAFLD, a global health problem [40]. Studies into causes and treatment of NAFLD have focused on *de novo* lipogenesis in liver overlooking the role of adipose tissue lipolysis. Lipolysis, particularly in visceral adipose tissue, may have a considerable impact on the liver due to the proximity of this depot to the hepatic portal vein [32] and because at least in humans visWAT lipolysis is highly responsive to catecholamines and less insulin sensitive than scWAT lipolysis [32]. Notably, a recent study identified PDE3B mutations that were associated with elevated blood TG levels and heart disease in humans [41].

The current study provides a major advance in our understanding of adipocyte lipolysis and its control by insulin. Our findings provide potential novel therapeutic avenues with which to tackle fatty liver as well as other diseases because dysregulated WAT lipolysis has been shown to contribute not only to fatty liver but also to hepatic insulin resistance [42], [43]. Hence it is not surprising that pharmaceutical inhibition of lipolysis in mice has beneficial effects on whole-body metabolism by lowering adiposity, NAFLD and insulin resistance [28], [29].

## Author's contributions

J.S. and D.E.J. conceived and designed the study. J.S. and A.Z. performed the majority of the experiments. K.C.C assisted with all animal and fat explant experiments. B.Y. and M.A.K. isolated islets and performed insulin secretion experiments. V.D. and S.J.H isolated adipocytes from white adipose tissue depots, performed MS analysis and MS data analysis. G.H. performed initial lipolysis experiments in 3T3-L1 adipocytes. J.S. and A.Z. performed data analysis and statistical analysis. J.S. and D.E.J. wrote the paper with help from all authors. All authors reviewed and edited the manuscript and approved the final version of the manuscript.
