# Supplementary material for: ABHD15 regulates adipose tissue lipolysis and hepatic lipid accumulation
Source: Mol Metab. 2019 May 6;25:83–94. doi: 10.1016/j.molmet.2019.05.002 (PMC6601125; doi:10.1016/j.molmet.2019.05.002)
Supplement: Multimedia component 1 [file mmc1.pdf]

Supplementary Table A

Protein.IDs	Gene.names	Chow_SC_1	Chow_SC_2	Chow_SC_3	Chow_VIS_1	Chow_VIS_2	Chow_VIS_3	WD_SC_1	WD_SC_2	WD_SC_3	WD_VIS_1	WD_VIS_2	WD_VIS_3
Q5F2F2;B6DQM2;F7C9J3;F6YMV	Abhd15	33.42	33.26	33.58	32.98	32.06	32.12	32.27	32.65	32.40	31.62	30.88	31.62
Q61409;E9QLQ3	Pde3b	32.78	32.63	32.42	32.19	32.24	32.37	32.00	31.97	31.59	31.07	31.23	30.73

Protein Intensity [log2]
